# Identification of Heterotic Groups and Patterns Based on Genotypic and Phenotypic Characteristics Among Rice Accessions of Diverse Origins

**DOI:** 10.3389/fgene.2022.811124

**Published:** 2022-01-28

**Authors:** Izhar Hussain, Sajid Ali, Wuge Liu, Muhammad Awais, Jinhua Li, Yilong Liao, Manshan Zhu, Chongyun Fu, Dilin Liu, Feng Wang

**Affiliations:** ^1^ Rice Research Institute, Guangdong Academy of Agricultural Sciences, Guangzhou, China; ^2^ Guangdong Provincial Key Laboratory of New Technology in Rice Breeding, Guangzhou, China; ^3^ Guangdong Rice Engineering Laboratory, Guangzhou, China; ^4^ Department of Plant Breeding and Genetics, The University of Haripur, Haripur, Pakistan; ^5^ Department of Agriculture, Hazara University Mansehra, Mansehra, Pakistan; ^6^ State Key Laboratory of Crop Stress Biology for Arid Areas, College of Plant Protection, Northwest A&F University, Yangling, China

**Keywords:** heterotic groups, heterotic patterns, hybrid rice, SNP, accession, genetic distance

## Abstract

Identification of the right parental combinations to maximize heterosis is the major goal of hybrid breeding, which could be achieved through identification of heterotic groups. The main objective of this study was to identify promising heterotic groups for future rice breeding programs. A collection of 359 rice genotypes of diverse origins of China and abroad, composed of inbreds, maintainers, restorers, and temperature-sensitive genic male sterile (TGMS) lines were genotyped using 10K SNP chips. The SNP data set was subjected to genomic analyses for estimation of genetic divergence and diversity. Significant variations were observed in the germplasm with the identification of six different genetic groups. These lines were assigned to the genetic groups independent of their origin. Taking an account of commercially used heterotic groups present in each cluster, three cytoplasmic male sterile (CMS) lines and 14 inbred and restorer lines with moderate to high genetic distances selected from five heterotic patterns were crossed and obtained 42 F_1_ hybrids. A total of 14 hybrids were found with significant maximum mid- and better-parent heterosis, namely, TaifengA × Guang122, TaifengA × Wushansimiao, and TaifengA × Minghui63 for earliness; Guang8A × Huazhan for dwarf stature; and Guang8A × Huanghuzhan-1, TaifengA × Yuexiangzhan, Guang8A × Minhui3301, TianfengA × Guang122, Guang8A × Yahui2115, TianfengA × Huanghuazhan, TianfengA × Minghui63, TianfengA × Minhui3301, TaifengA × Gui99, and Guang8A × Yuenongsimiao for yield and yield-related traits. Mid-parent and better-parent heterotic F1 hybrids were in positive correlation with the genetic distances as that manifested by commercially used heterotic groups, encouraging the use of genotypic data for identification of heterotic groups. Our study provides an informative strategy for the development of early maturing, lodging resistant and high-yielding commercial hybrids and cultivars in future heterosis breeding programs.

## 1 Introduction

Rice (*Oryza sativa* L.) is a staple food for over half of the world’s population. The continuous increase in rice consumption due to population increase (Khush, 2013) necessitates for higher rice production, which could be potentially achieved through rice genetic improvement. The development of hybrid varieties with high yield potential and resistance against disease and responsiveness to climatic changes could fulfill the future rice demands. In hybrid breeding, the most crucial element is identification of high-yielding heterotic patterns to achieve the maximum heterosis (Zhao et al., 2015). Genomic analyses could play a vital role in this regard. A heterotic group is a set of genetically related genotypes that show similar hybrid performance when crossed with individuals from another genetically distinct germplasm group (Melchinger and Gumber, 1984). Genetic relationship between genotypes of various accessions serves as one of the basic criteria for the outyielding potential of these heterotic groups (Thomson et al., 2008). The identification of heterotic groups in different germplasm pools is important for hybrid breeding (Xie et al., 2013; Wang et al., 2014). In general, the more divergent the heterotic groups are, the higher heterosis the offsprings have (Reif et al., 2005). Some studies, however, have reported the otherwise, which necessitates to include the phenotypic evaluation along with molecular marker data to explore both phenotypic and molecular diversity.

High genetic variations were detected in the Asian rice germplasm (Huang et al., 2012), which were divided into three *indica* subpopulations (South China origin, Southeast Asia origin, and IRRI inbred lines) and two japonica subpopulations (tropical and temperate; Wang et al., 2018a). The works on other groups like the aromatic rice have elucidated further diversity in the rice germplasm in different parts of the world (Civan et al., 2019). Identification of the heterotic groups among these various genetic stocks could be of immense importance for future hybrid breeding.

In hybrid rice crops, heterotic groups can be determined through marker-based genotyping (He et al., 2012). Molecular characterization of genetic diversity, population structure, and genetic relationships among breeding materials within a given set of genotypes will help to understand the use of the collected germplasm for further improvements, such as selecting parental lines and assigning to heterotic groups (Wu et al., 2016). So far, different kinds of molecular markers were used for diversity and divergence analyses in different species (Huang et al., 2012; Ali et al., 2016; Bueno-Sancho et al., 2017). Single-nucleotide polymorphism (SNP) is the most abundant and robust DNA sequence variation present in plant genomes, feasible for automated high-throughput genotyping and available for multiple assay options using different technology platforms to meet the demand for genetic studies and molecular breeding in crop plants (Steemers and Gunderson, 2007; Bernardo et al., 2009; Singh et al., 2015). Only superior parents do not necessarily produce superior heterotic combinations; rather, parents from different heterotic groups with high divergence (Reif et al., 2005) would give elite heterotic combinations (Zeng et al., 2007).

China is considered as the center of origin of *indica* rice and serves as a leading and major contributor of the world’s hybrid rice breeding (Cheng et al., 2007). Substantial diversity present in the region could be used to identify potential heterotic groups (Huang et al., 2012). Nowadays, the maintainer (sterile) lines and restorer lines have been derived from two major heterotic groups, widely used in the three-line indica hybrid rice breeding programs of China (Wang et al., 2006; Wang and Lu, 2006). The three-line system was first developed by Long Ping Yuan in the 1970s, which consists of a sterile restorer and a maintainer line (Yuan, 1986).

Presently, there has been little rigorous effort considering the genetic diversity and divergence for identification of the heterotic groups exploitable for hybrid rice development. Therefore, the present investigation was made to identify the heterotic groups based on genotypic characteristics of rice accessions of the South China origin, along with reference out group accessions from the United States, Philippines, Pakistan, Iran, and Thailand.

## 2 Materials and Methods

### 2.1 Plant Materials and DNA Extraction

A set of 352 *Indica* and seven *Japonica* genotypes were selected from different regions of China (Guangdong, Fujian, Guangxi, Hainan, Heilongjiang, Hubei, Hunan, Jiangsu, Jiangxi, Jilin, Sichuan, Yunnan, Taibei, Anhui, Chongqing, and Zhejiang), Philippines, United States, Pakistan, Iran, and Thailand. The set of these 359 lines was composed of 183 inbred lines, 53 maintainers, 120 restorers, one temperature-sensitive genic male sterile (TGMS) line, and two unidentified lines (Supplementary Table S1). These materials were used for genotyping through 10k SNP chips. The genomic DNA was extracted by cetyl trimethyl ammonium bromide (CTAB) method (Saghai-Maroof et al., 1984), and the quality and concentration of DNA were examined by agarose gel electrophoresis and Nano-Drop.

### 2.2 SNP Genotyping and *In Silico* Analysis of Sequence Data

We performed SNP genotyping *via* genotyping by target sequencing (GBTS) protocol GenoBaits, which is based on sequence capture in solution (also called a liquid chip). A 10K liquid rice chip developed by Mol Breeding Biotechnology Co., Ltd, Shijiazhuang, China was deployed. The protocol includes the steps of DNA library construction and probe hybridization, which was described in detail previously (Guo et al., 2019).

Sequence data generated by probe-in-solution target sequencing were subjected to in silico analysis as follows: the sequencing data were first checked for quality control; two-terminal reads were merged using FLASH, and sequencing data were then compared with the reference Nipponbare MSU 7.0 genome using BBMap. The alignment results were saved in the SAM/BAM (binary alignment map) format. SNP variants were detected from the BAM files using FreeBayes. The final variant calling was generated through GATK (2.4) (using Haplotype Caller in the gVCF mode) and joint genotyping (using Genotype GVCFs). The VCF file developed was filtered using criteria of MAF (minor allele frequency) > 0.05 and missing data > 80% at both the genotype and SNP marker levels. Only bi-allelic SNP markers with genotype quality > 20 and read depth > 5 were retained after using Vcftools v.0.1.12b (Danecek et al., 2011) and PLINK v1.07 (Purcell et al., 2007) for filtering.

### 2.3 Genomic Data Analyses

The final set of SNP data was subjected to genomic analyses for estimation of divergence and diversity. The genetic clusters were identified through discriminant analyses of principal component (DAPC) using the ADEGENET package implemented in R-software (Jombart et al., 2010). DAPC represents the non-parametric analyses which attempt to identify the genetic clusters without considering the origin of lines or their status as breeding lines (maintainer, restorer, etc.). Various numbers of clusters could be considered, and the lines were assigned to these clusters based on their genetic makeup. Thus, the DAPC analyses were run considering the possible clusters ranging from *K* = 2 to *K* = 10, where the most probable number of clusters was identified through the Bayesian Information Criteria (BIC) values (Jombart et al., 2010). The phylogenetic tree was constructed using the neighbor-joining method implemented in R-software based on their genetic distances, while the distribution of lines from the two ecotypes, various locations, and types of breeding lines was constructed in MEGA software. Information regarding diversity was estimated with POPPR applied on the GenLight object for populations defined based on ecotypes, locations of origin, and types of breeding lines (Kamvar et al., 2014). Genetic distances between heterotic groups were estimated through the Identity by Stat Distance Matrix method using TASSEL 5 software (Bradbury et al., 2007).

### 2.4 Plant Materials, Crossing, Field Experimentation, and Collection of Phenotypic Data

A total of 17 genotypes, composed of three maintainers, five inbreds, and nine restorer lines, were selected from five deduced heterotic groups (G-I, G-II, G-IV, G-V, and G-VI) on the basis of early maturity and high yielding performance with genetic distances ranging from 19.3 to 35.9% (Supplementary Table S6). In the late season of 2020, the three female lines, that is, TianfengA (C2330), TaifengA (C2230), and Guang8A (C2228), were crossed with the 14 male lines and obtained 42 new F1 hybrids. All the F1 hybrids and their parents were evaluated in Randomized Complete Block Design (RCBD) with three replications at Baiyun experimental base Guangzhou during early season of 2021. Observations were recorded on six earliness and yield-related traits, that is, days to 50% heading, plant height, panicles per plant, number of grains per panicle, 1,000-grain weight, and grain yield per plant.

### 2.5 Phenotypic Data Analyses

Analysis of variance was performed using Statistix 8.1. The mid parent and better parent (heterobeltiosis) were worked out as suggested by Dan et al. (2014) in Microsoft excel 2013. The correlation graphs of heterosis and genetic distances were also constructed in Microsoft excel 2013

$$\mathrm{Heterosis}\left(\%\right)\;=\;\;\frac{{\bar{F}}_{1}-\bar{MP}}{\bar{\mathrm{MP}}}\times \;100$$

$$\mathrm{Heterobelt iosis}\left(\%\right)=\;\;\frac{{\bar{F}}_{1}-\bar{\mathrm{BP}}}{\bar{\mathrm{BP}}}\times 100$$

## 3 Results

### 3.1 Summary Statistics on the SNPs

The 10,268 sites were evenly distributed on the short arm, centromere, and long arm of all the 12 chromosomes, as assessed for 359 rice genotypes. The number of SNPs on chromosomes 1, 2, 3, 4, 5, 6, 7, 8, 9, 10, 11, and 12 were 1,345, 1,097, 1,261, 914, 814, 899, 789, 672, 552, 573, 677, and 675, respectively. The average physical distance between SNPs is about 34.08 Kb based on a genome size of 350 Mb. The average minor allele frequency and the number of missing sites were 0.21989 and 0, respectively, whereas the proportion of heterozygous sites was 1.52% (Supplementary Table S2).

### 3.2 Diversity of the Breeding Population

**FIGURE 1:**
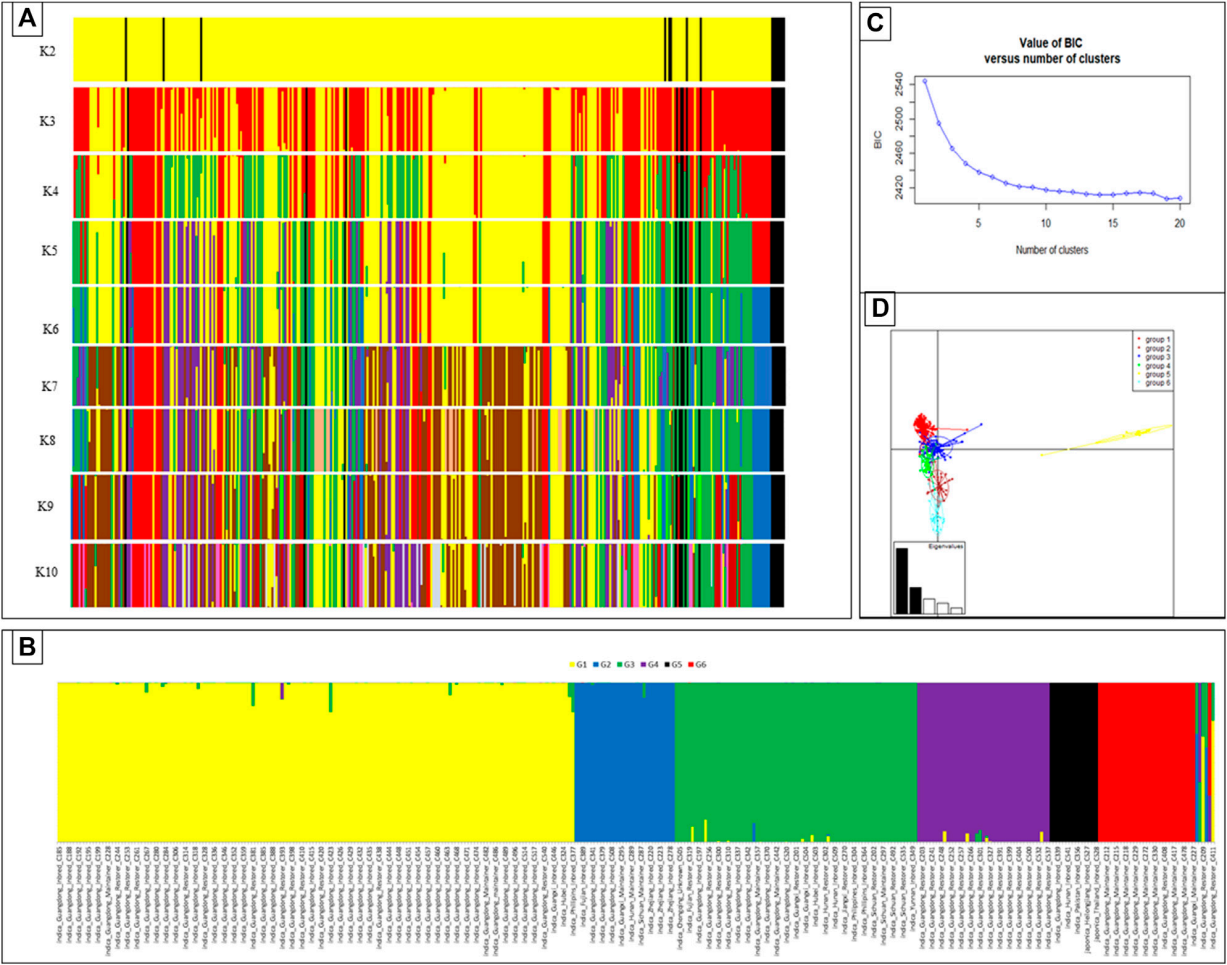
DAPC of rice accessions collected from different provinces in South China, Philippines, Thailand, Iran, Pakistan, and United States. Possible DAPC clusters ranging from K2 to K10 **(A)**. The cluster of 359 rice genotypes of diverse origins into different genetic groups set a siding geographical origin for the optimal K-value (*K* = 6) in DAPC **(B)**. Bayesian information criteria (BIC) supported six distinct genetic groups **(C)**. The Eigen values of the analysis suggest that the first two components explained the maximum genetic structure of the data set. Scatter plot of the 359 accessions divided into six genetic groups **(D)**.

Divergent groups were identified using discriminate analysis of principal components (DAPC) to represent potential diversity in the rice germplasm tested in this study. Grouping was made considering different K levels (K2-K10) of the DAPC analyses (Figure 1A). While considering the BIC values and principal component analysis grouping, six different genetic groups were considered the optimum within the rice germplasm (Figures 1B,C). In terms of the distribution of these genetic groups, G1 was dominant in the overall *Indica* germplasm, while the entire *Japonica* genotypes were grouped within a single group, that is, G5, with limited divergence among *Japonica* lines.

Considering the geographical origin, the most prevalent genetic group, that is, G1, contained most of the genotypes from the Guangdong origins, with a few genotypes from Guangxi, Hainan, Hubei, Hunan, Jiangxi, Sichuan, and Philippines present (≤3). Genetic group G2 was represented mainly by the lines from Zhejiang (all genotypes placed in this group), and group 3 contained all genotypes of the Yunnan origin along with few genotypes from diverse origins. Group G5 had all the genotypes of Heilongjiang, Jilin, Pakistan, and United States. A few of Guangdong and Jiangsu genotypes also belonged to this genetic group. Some of the Guangdong and Guangxi genotypes were assigned to group G6 (Figure 1B).

The distribution of the four types of breeding lines (inbred lines, maintainers, restorers, and the TGMS line) was also assessed to various genetic groups. DAPC results showed that the inbred lines, maintainers, and restorers were distributed across different genetic groups, and no genetic group was specific to any type of breeding lines. G1 was predominantly composed of the inbred lines, along with some maintainer and restorer lines. G6 was mainly represented by maintainer lines and very few restorers but no inbred lines. G3 was represented by all types of breeding lines, while G4 was represented by the restorers and a few maintainer lines (Figure 1B and Supplementary Table S3). This was in line with the cluster analysis-based grouping where all types were dispatched across different groups. Thus, all the genetic groups had substantial variability for these lines to be utilized for breeding purposes (Figure 1B and Supplementary Table S3).

### 3.3 Diversity Across Ecotypes and Breeding Lines

Low genetic diversity was recorded between groups (ranging from 0.144 to 0.303; Table 1). G’st values between groups (ranging from 0.324 to 0.427) indicated high divergence between heterotic groups suitable for future breeding programs. For all types of grouping patterns, the global heterozygosity value was 0.304. At the subspecies level, the highest value of 0.291 of diversity index was calculated for *Indica* subspecies containing 704 alleles, whereas a low diversity index of 0.107 was manifested by *Japonica* subspecies with 14 alleles only. The divergence calculated at subspecies level grouping was the maximum (0.548), as expected (Table 1). The number of alleles and diversity index in breeding lines ranged from 2 to 366 and from 0.003 to 0.304, respectively. Inbred lines showed the maximum value (0.304) of diversity index, followed by restorers (0.282), whereas the divergence value was the maximum (0.093) between breeding lines (Table 1). The genetic groups also revealed a high value of divergence (*G’st* = 0.427). Moreover, allelic frequencies of genetic groups ranged between 12 and 322, whereas the minimum value (12) was manifested by the group with unassigned lines and the maximum by group 1 (K1); however, the diversity index ranged between 0.182 and 0.303. Group 2 (K2) revealed the second highest value (0.244) of diversity index, followed by the group with unassigned lines (UN) with 0.243. K2 and K3 contained accessions from eight and 11 different locations, respectively, and thus had high diversity indices (Table 1).

**TABLE 1:** Amount of diversity index, heterozygosity, divergence, and number of alleles in ecotypes, breeding lines, locations, and genetic groups.

Grouping	Category	Sample size	Number of alleles	Diversity index	Total heterozygosity	Gst	G’st
Rice Ecotypes	Indica	352	704	0.291	0.304	0.095	0.548
	Japonica	7	14	0.107			
Breeding lines	Unknown	2	4	0.139	0.304	0.051	0.093
	Inbred	183	366	0.304			
	Maintainer	53	106	0.254			
	Restorer	120	240	0.282			
	TGMS	1	2	0.003			
Genetic Group	K1	161	322	0.202	0.304	0.324	0.427
	K2	31	62	0.244			
	K3	75	150	0.303			
	K4	41	82	0.191			
	K5	15	30	0.144			
	K6	30	60	0.182			
	UN	6	12	0.243			

### 3.4 Identification of Heterotic Groups

**FIGURE 2:**
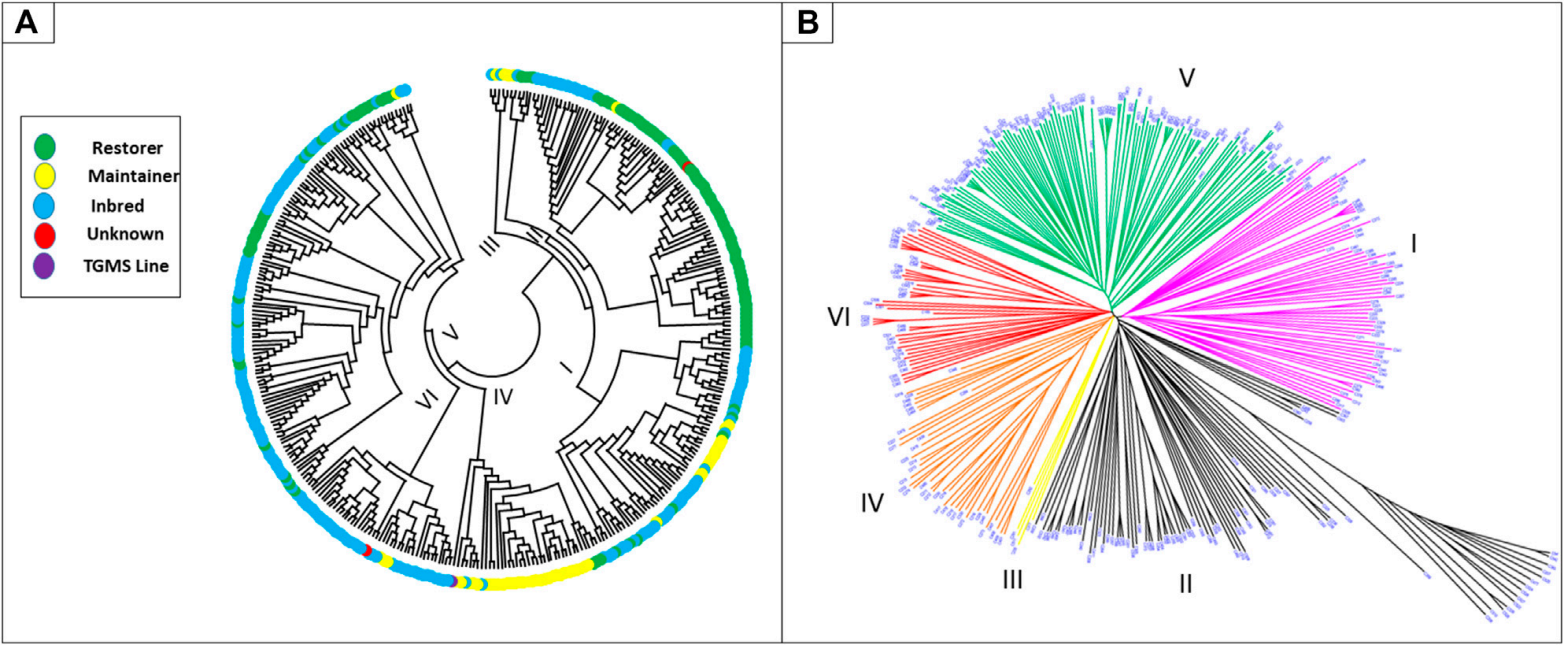
Distribution of breeding lines (inbreds, maintainers, restorers, TGMS, and unknown) into different clusters **(A)**. Phylogenetic tree, showing the overall distribution of 359 rice accessions into six different clusters **(B)**.

Genetic grouping was further confirmed *via* cluster analysis. The maximum number of accessions was recorded in cluster V (32.31%), followed by cluster II with (25.07%; Supplementary Table S4; Figure 2B). Similarly, cluster I contained 18.66% accessions in total, which was further divided into two subgroups, GI (12.3%) and GII (6.4%). Cluster II was further divided into four subgroups, GI (1.7%), GII (8.1%), GIII (4.2%), and GIV (11.1%). Cluster III was the smallest group that shared 1.39% of the accessions, whereas clusters IV and VI contained 10.6 and 11.14% of the total accessions, respectively (Supplementary Table S4).

*Indica* lines were clustered into six groups, while those of *Japonica* were located only in cluster II with high divergence from the rest accessions of the cluster (Figure 2B). The grouping of “*Indica*”-type rice lines in this group could be due to potential mismatches or erroneous labeling of these lines. Based on genetic information, inbred lines were dominant in clusters I, V, and VI; restorer lines were dominant in cluster II; and maintainer lines were dominant in clusters I and IV (Figure 2A and Supplementary Table S6). In cluster I, inbred lines (42), restorers (11), and maintainers (14) from 11 locations, and all the accessions from Zhejiang and Yunnan were present. The early developed and widely used maintainers, such as Zhenshan 97B (C288), BoB (C296), II-32B (C299), and the maintainer LongtefuB (C290) used in the development of high-yielding hybrids in South China, were also clustered into this group. Maintainer lines Gang46B (C368) and XiandangB (C293) were found very close to the commercial maintainer LongtefuB (C290) in cluster I (Figure 2B). Similarly, cluster II consisted of 19 inbreds, 69 restorers, and two maintainers from 14 locations. The most famous commercially used restorer lines Minghui63 (C281, C375), Guanghui998 (C203), and Gui99 (C536) were present in this group. Moreover, restorer lines R122 (C298), R308 (C251), R368 (C257), and R428 (C245), recently used for commercial hybrids, were also grouped in cluster II. The positions of restorer lines R998-3 (C533), R108 (C502), R122-3 (C537), Guang122 (C373), R721 (C303), R308-2 (C534), R390-1(C247), R290 (C299), R498 (C309), and R889 (C308) were close to the commercially used restorers. Cluster III was the smallest cluster with only two inbred (C377, C511) and three maintainer lines, which include the widely used maintainer 9311B (C235). Cluster IV was dominated by maintainers (32 out of 38 lines), including the widely used maintainers TianfengB (C330), WufengB (C272), RongfengB (C219), TaifengB (C230), HengfengA (C227), and Guang8B (C228). The maintainers in cluster IV are known as modern maintainer lines in China. Some other maintainer lines, such as JifengB (C217), WFB-TFB-derived (C418), and ZaofengB (C216), were closely related (Figure 2B) to the commercially used lines. Cluster V was composed of inbred lines (84) and restorers (32) but no maintainers. Although accessions from five different origins contributed to the cluster, the predominant location and breeding lines were Guangdong and inbred lines, respectively. Among the restorers in this cluster, Yuehesimiao (C190), R308 (C251), and Huazhan (C250) were widely used restorers. Using these commercially used lines as a close reference, we found three inbred lines, Yuehesimiao2 (C267), Guanghong3-3 (C538), and Yuexianzhan8 (C199), and two restorer lines, R721 (C303) and R308-2 (C534), which may also serve as heterotic group in the development of high-yielding hybrids. Moreover, cluster VI consisted of 40 inbreds and three maintainers from four locations. Similar to cluster V, Cluster VI also showed the greater contribution of inbred lines from Guangdong. The widely used maintainer YexiangB (C231) and the most famous aromatic Guangdong Simiao and the inbred varieties, Meixiangzhan 2 (C487), Xiangyaxiangzhan (C344), and Xiangzhuxiangsimiao (C428), were all placed in this cluster (Figure 2B). The presence of commercially used heterotic groups in all the six clusters indicated that we have six herterotic groups’ clusters in our germplasm.

### 3.5 Identification of Heterotic Patterns Between Groups

All the rice accessions have been divided into six clusters (heterotic groups), and the heterotic patterns could be deduced based on the accessions which served already as the parental lines of the heterotic hybrid combinations that existed, widely used for commercial production in China. It was as follows:

#### 3.5.1 Heterotic Pattern I (Cluster I × Cluster II)

Many famous maintainer lines, such as Zhenshan97B (C288), BoB (C296), LongtefuB (C290), and II-32B (C299), were located in Cluster I, while the famous restorer line Minghui63 (C281), R2156 (C263), R998 (C203), and Gui 99 (C201), were placed in Cluster II (Table 2 and Figure 2B). Many heterotic hybrids widely used for commercial production in China, such as Shanyou 63 (Zhenshan 97A/Minghui 63), Boyou 998 (BoA/R998), and ShanyouGui99 (Zhenshan 97A/Gui99), confirmed this pattern. It indicated that the hybrids derived from accessions of Cluster I and Cluster II had better heterosis; therefore, Cluster I and Cluster II could be a heterotic pattern. All the early-maturing inbred lines from Zhejiang Province and four accessions from Yunnan were located in Cluster I, which could be used for breeding new maintainer lines.

**TABLE 2:** Heterotic groups used for commercial hybrid production, genetic distance, and their deduced heterotic patterns.

Female parent (A)	Cluster	Male parent (R)	Cluster	Commercial hybrid	Genetic distance	Heterotic patterns
AnfengA (C478)	IV	Yuehesimiao (C190)	V	Antianyouyuehesimiao	0.351	IV × V
Guang8A (C228)	IV	Yuehesimiao (C190)	V	Guang8youyuehesimiao	0.277	IV × V
HengfengA (C227)	IV	Yuehesimiao (C190)	V	Hengfengyouyuehesimiao	0.290	IV × V
TaifengA (C230)	IV	Yuehesimiao (C190)	V	Taiyouyuehesimiao	0.285	IV × V
LongtepuA (290)	I	Yuehesimiao (C190)	V	Teyouyuehesimiao	0.294	I × V
TianfengA (C330)	IV	R122 (C298)	II	Tianyou122	0.341	IV × II
TianfengA (C330)	IV	R308 (C251)	V	Tianyou308	0.314	IV × V
TianfengA (C330)	IV	R368 (C257)	II	Tianyou368	0.309	IV × II
TianfengA (C330)	IV	R428 (C245)	II	Tianyou428	0.331	IV × II
WufengA (C272)	IV	Yuehesimiao (190)	V	Wuyouyuehesimiao	0.292	IV × V
Zhenshan97B (C288)	I	Minghui63 (C281)	II	Shan you 63	0.379	I × II
BoB (C296)	I	R998 (C203)	II	Boyou998	0.369	I × II
Zhenshan97A (C288)	I	Gui 99 (C536)	II	shan you Gui99	0.368	I × II
TianfengA (330)	IV	Guanghui 998(C203)	II	Tianyou 998	0.330	IV × II
WufengB (C272)	IV	R998 (C203)	II	Wuyou 998	0.325	IV × II
9311B (C235)	III	Huazhan (C250)	V	Quanyouhuazhan	0.304	III × V
RongfengB (C219)	IV	R463 (C269)	I	Rongyou 463	0.317	IV × I
Guang8A (C228)	IV	Yuenongsimiao (C265)	V	Guang8youyuenongsimiao	0.289	IV × V
Quan9311-A(235)	III	Wushansimiao (C320)	V	Quanyousimiao	0.310	III × V
Taifeng B(C230)	IV	R208(C248)	II	Rongyou Taiyou 208	0.325	IV × II
Jifeng B (C217)	IV	R1002 (C242)	II	Jifng you 1,002	0.335	IV × II
Tianfeng B(C330)	IV	Huazhan(C250)	V	Tian you huazhan	0.308	IV × V
Wufeng B(C272)	IV	R308(C251)	V	Wuyou 308	0.331	IV × V
Wufeng B(C272)	IV	Huazhan(C250)	V	Wuyouhuazhan	0.320	IV × V
Wufeng B(C272)	IV	Hanhui1179(C239)	V	Wuyou1179	0.314	IV × V
Tianfeng B (C330)	IV	R305(C381)	V	Taiyou 305	0.327	IV × V
Tianfeng B (C330)	I	R398 (C243)	IV	Taiyou 398	0.291	I × IV
Jifeng B (C217)	I	V1100(C300)	IV	Jiyou 1,100	0.317	I × IV
Te B (C290)	I	R721(C303)	V	Teyou 721	0.315	I × V
YexiangB (C231)	VI	Fuhui 676 (C319)	II	Yexiangyou 676	0.315	VI × II
				Mean	0.319	
				Minimum	0.277	
				Maximum	0.379	

#### 3.5.2 Heterotic Pattern II (Cluster IV × Cluster II)

A number of super hybrid rice varieties were derived from this crossing pattern, including Tianyou998 from TianfengB (C330) and R998 (C203), Wuyou998 from WufengB (C272) and R998 (C203), Taifengyou 208 from TaifengB (C230) and R208 (C248), and Jifengyou 1,002 from JifengB (C217) and R1002 (C242). All the female parents of these hybrids were taken from cluster IV, and male parents were taken from cluster II (Table 2).

#### 3.5.3 Heterotic Pattern III (Cluster IV × Cluster V)

The super rice hybrid “Tianyouhuazhan” was derived from TianfengB (C330) and Huazhan (C250), “Wuyou308” from Wufeng B (C272) and R308 (C251), Wuyouhuazhan from WufengB (C272) and Huazhan (C250), Wuyou1179 from WufengB (C272) and Hanghui1179 (C239), and Taiyou305 from TaifengB (C230) and R305 (C381), all supporting this heterotic group pattern.

#### 3.5.4 Heterotic Pattern IV (Cluster III × Cluster V)

The famous hybrid Quanyousimiao was derived from 9311B (C235) and Wushansimiao (C320), and Quanyouhuazhan was derived from 9311B (C235) and Huazhan (C250), supporting this heterotic pattern.

#### 3.5.5 Heterotic Pattern V (Cluster IV × Cluster I)

The widely planted early-maturing hybrid Taiyou398 derived from TaifengB(C230) and R398 (C243) and Jiyou 1,100 derived from Jifeng B (C217) and V1100 (C300) supported this pattern.

#### 3.5.6 Heterotic Pattern VI (Cluster I × Cluster V)

The high-yielding hybrid rice hybrid Teyou 721 derived from LongtefuB (C290) and R721 (C303) supported this pattern.

#### 3.5.7 Heterotic Pattern VII (Cluster VI × Cluster II)

The fine-quality hybrid Yexiangyou 676 supported this pattern as it was derived from YexiangB (C231) and Fuhui676 (C319).

All the six Clusters I–VI had already been involved in the seven heterotic patterns mentioned above, so these clusters could be considered as heterotic groups.

### 3.6 Heterotic Group and Genetic Distance

The diversity analyses of DAPC-based groups revealed significant diversity for all the heterotic groups, that is, K1 (0.202), K2 (0.244), K3 (0.303), K4 (0.191), K5 (0.144), K6 (0.182), and UN (0.283; Table 1). Similarly, the G’st value (0.427) also summarized the overall mean diversity (distances) between the heterotic groups, which were at the optimum level (Table 1). Genetic distances between the heterotic groups, the deduced heterotic groups of commercially used hybrids, and their nearby heterotic groups spotted on the Neighbor Joining tree (Figure 2B) were estimated through the Identity by state (IBS) matrix ranging between 0.01 and 0.391 with a mean value of 0.276 (Supplementary Table S5). The maximum genetic distance (0.391) was observed for the heterotic group Gang46B (C368) × R498 (C309), followed by Gang46B (C368) × Minghui63 (C281) (0.389), whereas the minimum genetic distance (0.010) was noted for R301-1 (C251) × R308-2 (C534), followed by (0.013) R998-3 (C533) × R998-1 (C203). However, the majority groups were found very close to the average (0.28) genetic distances (Supplementary Table S5). Among commercially used heterotic groups, the maximum genetic distance (0.379) was observed for Zhenshan 97B (C288) × Minghui63 (C281) of the deduced heterotic pattern (GI ×GII), followed by Bo B (C296) × R998 (C203) and Zhenshan 97A (288) × Gui 99 (536) with genetic distances of 0.369 and 0.368, respectively, from heterotic patterns (GI × GII) (Table 2). Majority of the commercially used heterotic groups showed greater genetic distances than the overall mean genetic distance of 0.276, which reflected that the genetic distances between heterotic groups have a positive effect on heterosis.

### 3.7 Variability for Earliness and Yield-Related Phenotypic Traits in F1 Hybrids and Their Parents

Analysis of variance revealed highly significant (*p* < 0.01) differences among genotypes for days to 50% heading, plant height, panicles per plant, number of grains per panicle, 1,000-grain weight, and grain weight per plant (Table 3; Supplementary Table S7). Days to 50% heading ranged from 76.33 to 101.67 days, with a net difference of 25.34 days (Supplementary Table S8). The plant heights varied from 88.00 to 131.00 cm, with a net difference of 43 cm and a majority of the F_1_ hybrids close to the mean (110.60 cm). The mean values of 4.33 to 11.00 panicles per plant were observed among genotypes. Almost all F_1_ hybrids revealed above-average performance for panicles per plant. For grains per panicle, the genotypes ranged from 30.41 to 173.61, showing a wide range of variability. A majority of hybrids showed above-average performance, and none of the hybrids was observed at par to the minimum. The mean values for 1,000-grain weight varied from 20.893 to 32.013 g. For grain yield per plant, the mean values of the genotypes ranged between 9.48 and 40.35 g, among which the maximum grain yield was produced by the three hybrids Guang8A × Yuenongsimiao (40.350 g), TaifengA × Gui99 (36.480 g), and TaifengA × Guang122 (35.250 g), followed by two other F_1_ hybrids, TaifengA × Minghui63 (31.537 g) and TaifengA × Huanghuazhan (30.823 g).

**TABLE 3:** Analysis of variance for earliness, plant height, yield, and yield-related traits.

S.No	Traits	GMS	Ems	F-ratio	CV (%)
1	Days to 50% heading (#)	120.63	1.13	106.54***	1.19
2	Plant height (cm)	192.77	5.38	35.82***	2.12
3	Panicles per plant (#)	6.42	0.44	14.61***	8.95
4	Grains per panicle (#)	2,222.70	175.78	12.64***	10.93
5	1,000-grains weight (g)	59.06	0.08	743.82***	1.09
6	Grains weight per plant (g)	100.44	2.97	33.85***	7.52

### 3.8 Heterosis Estimates on the Basis of Phenotypic Performance

Heterosis over the mid parent and the best parent (heterobeltiosis) was studied in 38 F_1_ hybrids for various traits. For heading date, significant negative heterosis over mid and better parents was exhibited by nine and two F_1_ hybrids, respectively. Negative heterosis over the mid parent ranged from −0.36% (Guang8A × Huanghuazhan-1) to −9.49% (TaifengA × Guang122), whereas ranging from 0.55% (Guang8A × Wushansimiao) to 10.02% (TianfengA × Huanghuazhan), 18 F_1_ hybrids manifested mid parent-positive heterosis (Table 4). The better-parent heterotic performance ranged from −1.47% (Guang8A×Huazhan) to −2.97% (TaifengA × Guang122). Better-parent significantly positive heterosis ranged between 1.83% (Guang8A × Huanghuazhan-1) and 27.57% (TianfengA × Minhui3301). For heading date, negative heterosis is favored because it leads to earliness. A total of 19 F_1_ hybrids showed mid-parent heterosis and three F_1_ hybrids showed better-parent heterosis with negative values, in which nine mid parents and two better parents reached a significance level. Plant height revealed low to moderate levels of positive mid- and better-parent heterosis for a majority of F_1_ hybrids. One F1 hybrid (Guang8A × Huazhan) showed negative mid-parent and better-parent heterosis (−4.04%). A total of 10 F_1_ hybrids revealed positive mid-parent heterosis for panicles per plant, and maximum heterotic values were exhibited by the F_1_ hybrid Guang8A × Huanghuazhan-1 (31.30%). Only one F_1_ hybrid (Guang8A × Huanghuazhan-1) showed positive heterobeltiosis (14.24%) for panicles per plant. However, the remaining F_1_ hybrids manifested negative heterosis over the better parent (Table 4).

**TABLE 4:** Mid-parent and better-parent heterosis estimates for days to 50% heading, plant height, panicles per plant, and genetic distances between their corresponding parents.

Cross code	F1 hybrid name	Days to 50% heading		Plant height		Panicles per plant		GD
		MPH	BPH	MPH	BPH	MPH	BPH	
C330 × C373	TianfengA × Guang122	4.55**	18.22**	16.99**	20.83**	−13.31**	−20.2**	0.34
C330 × C205	TianfengA × Huanghuazhan-1	2.61**	19.63**	6.53**	17.55**	−10.3	−22.8**	0.3
C330 × C250	TianfengA × Huazhan (HZ)	5.95**	20.56**	12.81**	23.99**	−22.59**	−24.1**	0.31
C330 × C375	TianfengA × Minghui63	2.50**	24.3**	16.00**	30.43**	−4.99	−15.2**	0.35
C330 × C472	TianfengA × Wushansimiao	4.56**	17.76**	5.71**	21.59**	1.24	0	0.29
C330 × C447	TianfengA × Huanghuazhan	10.02**	25.7**	7.74**	16.92**	−32.45**	−35.5**	0.24
C330 × C268	TianfengA × Minhui3301	3.02**	27.57**	17.26**	31.69**	−0.01	−25.3**	0.35
C330 × C493	TianfengA × Chenghui727	6.75**	25.7**	10.65**	27.28**	−17.30**	−30.4**	0.34
C330 × C492	TianfengA × Yahui2115	9.02**	27.1**	9.40**	34.47**	−26.48**	−36.7**	0.36
C330 × C201	TianfengA × Gui99	4.72**	19.16**	8.18**	21.97**	4.7	−1.28	0.34
C330 × C282	TianfengA × Yuexiangzhan	3.79**	21.5**	13.75**	30.56**	−10.24	−27.9**	0.31
C330 × C386	TianfengA × Yuenongsimiao	7.16**	22.43**	9.56**	21.59**	−0.03	−17.7**	0.27
C230 × C373	TaifengA × Guang122	−9.49**	−2.97**	5.39**	7.82**	−29.29**	−31.9**	0.33
C230 × C205	TaifengA × Huanghuazhan-1	−0.96	9.32	4.30**	8.61**	5.57	−12.6**	0.31
C230 × C250	TaifengA × Huazhan (HZ)	−2.95**	4.66**	3.82**	7.7**	−11.66*	−17.2**	0.29
C230 × C375	TaifengA × Minghui63	−7.58**	5.93**	6.09**	12.46**	14.07**	−2.31	0.33
C230 × C472	TaifengA × Wushansimiao	−8.73**	−2.54**	0.36	8.72**	−3.57	−6.9	0.31
C230 × C447	TaifengA × Huanghuazhan	−0.59	7.63	0.66	3.17*	−8.2	−16.1**	0.24
C230 × C268	TaifengA × Minhui3301	−5.07**	11.02**	11.55**	18.12**	20.63**	−12.6**	0.32
C230 × C493	TaifengA × Chenghui727	−0.38	11.02	11.03**	20.27**	6.38	−13.8**	0.31
C230 × C492	TaifengA × Yahui2115	−1.34	8.9	3.92**	19.93**	−13.89*	−28.7**	0.33
C230 × C201	TaifengA × Gui99	−1.38	6.36	0.58	6.91**	−0.63	−10.3*	0.32
C230 × C282	TaifengA × Yuexiangzhan	−2.10**	8.47**	1.2	9.4**	28.89**	0	0.32
C230 × C386	TaifengA × Yuenongsimiao	−2.54**	5.51**	1.57	6.34**	−17.41**	−34.5**	0.29
C228 × C373	Guang8A × Guang122	2.76**	2.2**	8.33**	1.99**	−26.33**	−33**	0.27
C228 × C205	Guang8A × Huanghuazhan-1	−0.36	1.83*	5.65**	5.65**	31.30**	14.24**	0.23
C228 × C250	Guang8A × Huazhan (HZ)	−1.47	−1.47	−4.04**	−4.39**	−39.86**	−40.3**	0.22
C228 × C375	Guang8A × Minghui63	−1.73**	4.03**	6.02**	7.85**	−9.37	−18.2**	0.31
C228 × C472	Guang8A × Wushansimiao	0.55	1.49	−1.91	1.89	−24.06**	−25.9**	0.19
C228 × C319	Guang8A × Fuhui676	5.54**	11.72**	7.35**	19.88**	−29.89**	−39**	0.29
C228 × C447	Guang8A × Huanghuazhan	0.73	1.1	3.77**	2.2	−10.05	−13*	0.22
C228 × C268	Guang8A × Minhui3301	−3.57**	4.03**	6.64**	8.37**	24.13**	−6.51	0.29
C228 × C493	Guang8A × Chenghui727	−0.89	2.2**	−0.3	3.56*	2.28	−13*	0.29
C228 × C492	Guang8A × Yahui2115	−1.43	0.73	3.08**	13.81**	−19.41**	−29.9**	0.33
C228 × C201	Guang8A × Gui99	0	0	2.26	4.29**	−15.65**	−19.5**	0.28
C228 × C307	Guang8A × Ce64	2.36**	3.3**	10.48**	3.14*	−25.00**	−33.3**	0.28
C228 × C282	Guang8A × Yuexiangzhan	−1.07	1.47	7.67**	11.61**	−15.21*	−31.2*	0.24
C228 × C386	Guang8A × Yuenongsimiao	2.55**	2.93**	3.12**	3.66*	−17.21**	−31.2**	0.21
	Mean	0.67	9.96	6.40	13.47	−8.29	−20.10	0.29
	Minimum	−9.49	−2.97	−4.04	−4.39	−39.86	−40.30	0.19
	Maximum	10.02	27.57	17.26	34.47	31.30	14.24	0.36

More than half of the F_1_ hybrids depicted significant positive mid-parent heterosis for grains per panicle (Table 5). Heterotic effects varied from 17.49% (Guang8A × Huanghuazhan) to 74.39% (Guang8A × Minghui63) over their mid parents. Significantly positive better-parent heterosis was recorded on 14 F_1_ hybrids, ranging from 16.73% (TianfengA × Minhui3301) to 45.01 (Guang8A × Yahui2115). A majority of the F_1_ hybrids showed significant positive mid- and better-parent heterosis for 1,000-grain weight (Table 5). The F_1_ hybrids TaifengA × Minghui63 and TaifengA × Minhui3301 revealed the highest values of 20.37 and 17.98% over their mid- and better-parental inbred lines, respectively. Regarding mid-parent heterosis for grain yield per plant, 18 and 15 F_1_ hybrids manifested significant positive mid- and better-parent heterosis, respectively (Table 5). Mid-parent significantly positive heterosis ranged from 20.73% (TianfengA × Minhui3301) to 94.99% (TaifengA × Gui99). The latter promising F_1_ hybrid was followed by four other high-yielding hybrids, Guang8A × Yuenongsimiao (77.00%), TaifengA × Huanghuazhan-1 (71.19%), TaifengA × Guang122 (60.08%), and TaifengA × Minghui63 (59.01%). For better-parent heterosis, the F_1_ hybrid TaifengA × Gui99 (71.03%) exhibited the most significant positive heterotic effects.

**TABLE 5:** Mid-parent and better-parent heterosis estimates for grains per panicle, 1,000-grain weight, grain weight per panicle, and genetic distances between their corresponding parents.

Cross code	F1 hybrid name	Grains per panicle		1,000-grain weight		Grain weight per plant		GD
		MPH	BPH	MPH	BPH	MPH	BPH	
C330 × C373	TianfengA × Guang122	60.06**	33.85**	13.25**	8.58**	11.1	7.88**	0.34
C330 × C205	TianfengA × Huanghuazhan-1	23.07**	17.79*	−1.98**	−9.73**	45.34**	22.53**	0.3
C330 × C250	TianfengA × Huazhan (HZ)	13.00	12.03	7.16**	−3.61**	−47.86**	−43.2**	0.31
C330 × C375	TianfengA × Minghui63	62.23**	2.77	16.40**	14.05**	34.61**	25**	0.35
C330 × C472	TianfengA × Wushansimiao	5.03	4.77	8.70**	1.19	52.45**	20.2*	0.29
C330 × C447	TianfengA × Huanghuazhan	39.58**	32.63**	6.57**	−0.15	21.95*	21.01*	0.24
C330 × C268	TianfengA × Minhui3301	50.48**	16.73*	18.63**	15.14**	20.73*	14.7	0.35
C330 × C493	TianfengA × Chenghui727	19.54*	12.91	5.99**	−1.3*	26.13**	14.05	0.34
C330 × C492	TianfengA × Yahui2115	29.25**	23.98**	6.46**	6.33**	4.04	3.08	0.36
C330 × C201	TianfengA × Gui99	−3.41	−4.69	2.96**	1.99**	−20.94*	−30.8**	0.34
C330 × C282	TianfengA × Yuexiangzhan	18.26*	11.33	9.18**	2.5**	9.25	2.23	0.31
C330 × C386	TianfengA × Yuenongsimiao	5.21	−12.6*	1.75*	−4.6**	49.39**	32.15**	0.27
C230 × C373	TaifengA × Guang122	37.44**	27.57**	7.86**	7.28**	60.08**	55.22**	0.33
C230 × C205	TaifengA × Huanghuazhan-1	18.10*	9.78*	−8.05**	−18.9**	71.19**	44.51**	0.31
C230 × C250	TaifengA × Huazhan (HZ)	13.54	2.13	16.43**	9.46**	−11.76	−20.9**	0.29
C230 × C375	TaifengA × Minghui63	73.99**	16.57	20.37**	17.11**	59.01**	37.86**	0.33
C230 × C472	TaifengA × Wushansimiao	14.93	2.82	7.66**	4.93**	−43.67**	−55.5**	0.31
C230 × C447	TaifengA × Huanghuazhan	−11.34	−24.3**	6.37**	4.39**	23.41**	22.64**	0.24
C230 × C268	TaifengA × Minhui3301	60.62**	36.71**	20.11**	17.98**	10.32	4.67	0.32
C230 × C493	TaifengA × Chenghui727	3.54	−2.5	12.30**	0.11	3.47	−6.31	0.31
C230 × C492	TaifengA × Yahui2115	34.40**	24.65**	8.87**	3.97**	25.88**	24.54**	0.33
C230 × C201	TaifengA × Gui99	11.12	−1.96	4.61**	−1.12	94.99**	71.03**	0.32
C230 × C282	TaifengA × Yuexiangzhan	10.73	−6.25	9.61**	7.79**	8.01	0.92	0.32
C230 × C386	TaifengA × Yuenongsimiao	21.65**	−7.58	5.63**	3.73**	27.29**	12.75	0.29
C228 × C373	Guang8A × Guang122	43.94**	20.37**	2.93**	−1.86*	−6.54	−14	0.27
C228 × C205	Guang8A × Huanghuazhan-1	−6.02	−10.1	−4.74**	−19**	27.23*	12.52	0.23
C228 × C250	Guang8A × Huazhan (HZ)	40.59**	39.38**	−0.29	−2.29**	−9.66	−22.8**	0.22
C228 × C375	Guang8A × Minghui63	74.39**	10.48	9.70**	2.42**	17.85	15.52	0.31
C228 × C472	Guang8A × Wushansimiao	8.38	8.11	6.29**	4.48**	20.84	−0.52	0.19
C228 × C319	Guang8A × Fuhui676	53.68**	23.17**	6.92**	−0.1	36.89**	24.58*	0.29
C228 × C447	Guang8A × Huanghuazhan	17.49*	11.64	−4.55**	−6.82**	41.11**	34.5**	0.22
C228 × C268	Guang8A × Minhui3301	15.7	−10.3	5.58**	−0.53	−7.18	−16.3*	0.29
C228 × C493	Guang8A × Chenghui727	12.05	5.83	−2.12**	−15.9**	41.43**	34.77**	0.29
C228 × C492	Guang8A × Yahui2115	51.16**	45.01**	1.95*	−6.5**	16.97	9.72	0.33
C228 × C201	Guang8A × Gui99	20.31**	18.72*	−1.90*	−10.9**	−1.7	−9.44	0.28
C228 × C307	Guang8A × Ce64	44.50**	29.73**	0.14	−3.3**	9.87	0.74	0.28
C228 × C282	Guang8A × Yuexiangzhan	13.29	6.65	10.98**	8.12**	5.67	−6.08	0.24
C228 × C386	Guang8A × Yuenongsimiao	21.36**	0.81	−0.01	−2.46**	77.00**	60.33**	0.21
	Mean	26.89	11.28	6.26	0.854	21.16	10.63	0.29
	Minimum	−11.34	−24.30	−8.05	−19.00	−47.86	−55.50	0.19
	Maximum	74.39	45.01	20.37	17.98	94.99	71.03	0.36

### 3.9 Genetic Distance Effects on Heterosis

**FIGURE 3:**
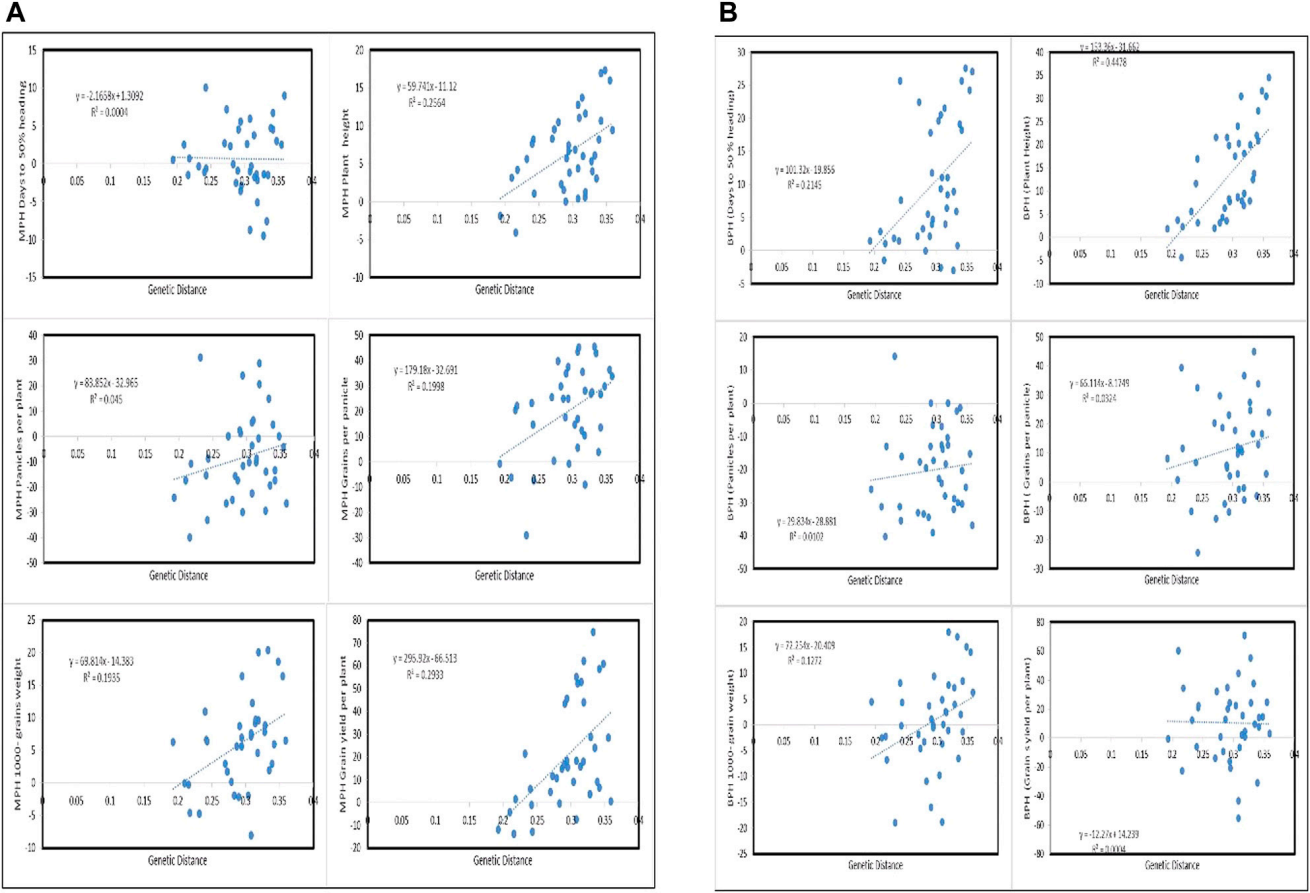
Representation of mid-parent **(A)** and better-parent **(B)** heterosis association of F1 hybrids with the genetic distances between their corresponding parents.

Genetic distances between the parents of 38 F_1_ hybrids were estimated through IBS in TASSEL 5, which ranged between 19.00 and 36.00% (Table 5). For days to 50% heading, F_1_ hybrids of significant mid-parent-negative and mid-parent-positive heterosis manifested slightly negative correlation with genetic distances, whereas better-parent heterosis showed positive correlation with the genetic distances (Figure 3B). Similarly, heterosis over mid and better parents for plant height, panicles per plant, number of grains per panicles, and 1000-grain weight also showed positive association with the genetic distances. A majority of the F_1_ hybrids with highly significant heterosis were present at the maximum end of genetic distances (Figures 3A,B). Mid-parent heterosis for grain yield per plant in F_1_ hybrids was found in positive correlation with the genetic distances in their corresponding parents, whereas better-parent heterosis was observed in slightly negative correlation with genetic distances (Figures 3A,B).

## 4 Discussion

Despite the success of hybrid rice since 1970s (Lin et al., 2020), the understanding of the heterosis group and heterotic pattern in rice is very limited (Wang et al., 2014). The maximum benefit out of the heterotic vigor could be achieved through the assessment of diversity and divergence in the rice germplasm for identification of the potential heterotic groups, for which high-throughput genotyping is of great help (Zhao et al., 2011; Wang et al., 2014; Wang et al., 2018b). In this study, heterotic groups were identified using a 10K SNP chip, in different *Indicia* and *Japonica* genotypes selected from different origins of China and abroad, including 183 inbred lines, 53 maintainers, 120 restorers, one TGMS line, and two unknowns.

Divergence analyses revealed the existence of six subgroups among subspecies, breeding lines, origins, and genetic groups. Up to K6, DAPC-based grouping was stable and was supported by both PCA and BIC analyses. Along with the overall variability in the tested germplasm, substantial variability was observed in each genetic group, geographically collected lines, and breeding lines. Geographical distribution of lines could also contribute to the existence of subgroups (Zhang et al., 2011). However, in this study, a no-population subdivision was observed due to geography/locations, except that lines from two regions, that is, Zhejiang and Yunnan, were grouped only in a single group (G2 and G3, respectively). Effective evaluation of diversity provides a considerable scope of choice of parents before hybridization (Pandey et al., 2011). Phylogenetic analysis showed that genotypes obtained from different origins had significant variation and were assigned into different groups. Huang et al. (2012) also find out a large-scale genetic variation in the Asian cultivated rice germplasm. Moreover, cluster analysis also confirmed that there are six different clusters, and the maintainers were distributed in three independent clusters. Almost similar principal components were identified in previous studies (Rathnathunga and Geekiyanage, 2015, 2016, 2017; Rathnathunga et al., 2016), which recommended variable levels of diversity in various rice germplasms.

Estimation of phenotypic and genotypic diversity provides useful information for the establishment of heterotic patterns (Agre et al., 2019). As all the six clusters contained the commercially used high-yielding parents, each cluster was considered as the basis for heterotic groups. From all commercially used hybrids and new combinations, seven heterotic patterns were identified. The higher genetic distance among the commercially used lines reflected positive association with heterosis; thus, new heterotic groups with higher genetic distances could be predicted. As suggested previously, significant differences among the rice genotypes were expected to provide better hybrid vigor (Prasanna et al., 2010; Mvuyekure et al., 2018).

Following the heterotic pattern of cluster IV × cluster II, the best modern maintainer lines in cluster IV could be used with the best restorers of cluster II in the development of high-yielding hybrids. Elite inbred lines from cluster V can be used as male parents of two lines and three lines of hybrid rice. Moreover, Cluster V was predominated by inbred cultivars of the Guangdong origin, which usually have good grain quality and better resistance to rice blast and bacterial blight, and is suitable to be deployed in breeding new restorer lines.

Considering the aforementioned findings, 14 inbred and restorer lines from groups I, II, V, and VI were crossed with three CMS lines from group IV. The mean performance for various parameters revealed a substantial variability. The F_1_ hybrids obtained from the partial diallel crosses and their parents revealed significant variations for all the studied traits, which can provide an ample scope for further improvement. A majority of the F_1_ hybrids showed higher mean performance than their parents. In agreement with our study, significant variation for yield and yield-related traits among rice genotypes was observed previously (Singh et al., 2006; Prasad et al., 2013; Ganapati et al., 2014; Asem et al., 2019).

Heterosis is critical for the estimation and development of new plant population (Cheng et al., 2019; Venkatesan et al., 2019; Rasheed et al., 2021). Although the overall heterosis for heading date and plant height was at low and moderate levels, some TaifengA progenies and Guang8A × Huazhan for plant height manifested significant negative heterosis over the mid and best parents, similar to other studies (Selvaraj et al., 2011; Kumar et al., 2012). Four F_1_ hybrids of TaifengA showed significant earliness and can be used for developing early maturing and lodging-resistant dwarf stature hybrids. The number of panicles per plant also showed moderate levels of significant positive heterosis, where the F_1_ hybrids such as TaifengA × Minhui3301 exhibited maximum heterosis. Corroborating results of similar nature heterosis were reported (Gnanamalar and Vivekanandan, 2013; Rukmini et al., 2014; Lingaiah et al., 2019). Quantitative traits, that is, grain weight, grain number per panicle, and number of panicles, positively contribute to the yield (Rasheed et al., 2021). High levels of mid- and better-parent heterosis were found for the number of grains per panicle, and all the three maternal lines showed significant heterotic effects with different inbred and restorer lines in our study, which is in accordance with the previous findings (Priyanka et al., 2014; Lingaiah et al., 2019). Similarly, for 1,000-grain weight, which is one of the key components of yield, F1 hybrids TaifengA × Minghui63 and TianfengA × Minhui3301 were found with moderately significant positive heterosis over the mid and better parents, and TaifengA × Minhui3301, TianfengA × Guang122, and TaifengA × Huazhan showed mid-parent heterosis and TianfengA × Minghui63 manifested heterobeltiosis only. Mostly significant positive mid- and better-parent heterotic performances were recorded for 1,000-grain weight (Lingaiah et al., 2019). In the case of grain yield per plant, a majority of F_1_ hybrids were found with above-average positive heterosis. High levels of significant positive heterosis over mid and better parents were manifested by F_1_ progenies such as TaifengA × Gui99, which can be used as potential sources for the development of high-yielding hybrids in future breeding. Advocating results of high heterosis over mid parents and better parents were reported previously (Zhang et al., 1994; Alzona and Arraudeau, 1995).

In the present study, a majority of mid-parent and best-parent heteroses were with positive association with genetic distances. Except mid-parent heterosis for days to 50% heading and better-parent heterosis for grain yield per plant, which were found in slightly negative correlation with genetic distances, all the studied traits exhibited positive correlation with the genetic distances. Considering the genetic variation as a source for heterotic gain, several studies were conducted to unveil the relationship between genetic distances and heterosis for predicting the heterosis effect and found that to some extent, heterosis is positively associated with genetic distances (Lee et al., 1989; Smith et al., 1997; Zhao et al., 2009). Although greater achievement of hybrid breeding depends on the identification of complementary heterotic groups (Reif et al., 2007; Zhao et al., 2015), the heterotic groups in rice are still not clearly defined (Xie et al., 2012). Corroborating results were obtained by maximizing the genetic distances for separation of maize lines into groups, showing the advantage of a significant yield over within-group crosses. Thus, the groups estimated by increasing the genetic distances could be a meaningful source for heterotic group development (Suwarno et al., 2014). Wang et al. (2014) estimated the magnitude of yield heterosis among selected heterotic groups with greater genetic distances and observed that hybrids had more yield than their parents, with an average of 24.1% mid-parent heterosis, which is in line with our findings. Similarly, the molecular marker approach was used to estimate the genetic distances between breeding lines for dividing the germplasm into heterotic groups (Prasanna et al., 2010). Singh et al. (2015) also estimated the genetic diversity and phylogenetic relationship among 128 diverse rice germplasms using 50K rice SNP chips. Haplotype analysis separated the 128 genotypes into four major heterotic groups, revealing that the genotypes are grouped on the basis of their genetic makeup (genetic distances).

## 5 Conclusion

In conclusion, considering the mid-parent and better-parent significant heterosis and promising mean performance, our results have identified 14 heterotic combinations, that is, TaifengA × Guang122, TaifengA × Wushansimiao, and TaifengA × Minghui63 for earliness; Guang8A × Huazhan for dwarf stature; and Guang8A × Huanghuzhan-1, TaifengA × Yuexiangzhan, Guang8A × Minhui3301, TianfengA × Guang122, Guang8A × Yahui2115, TianfengA × Huanghuazhan, TianfengA × Minghui63, TianfengA × Minhui3301, TaifengA × Gui99, and Guang8A × Yuenongsimiao for yield and yield-related traits. F1 hybrid heterosis over the mid and better parents was in positive correlation with the genetic distances. These F1 Hybrids should be used in the development of early-maturing, lodging-resistant, and high-yielding commercial hybrids and cultivars in future heterosis breeding programs after multilocation and multiyear testing. The use of genetic distance must complement with phenotypic characterization for identification of heterotic groups and generation of promising hybrids.

## Data Availability

The original contributions presented in the study are publicly available. This data can be found here: The variation data reported in this paper have been deposited in the Genome Variation Map (GVM) (Song et al., 2018) in Big Data Center (BIG Data Center Members, 2018), Beijing Institute of Genomics (BIG), Chinese Academy of Science, under accession numbers GVM000300 at http://bigd.big.ac.cn/gvm/getProjectDetail?project=GVM000300.
