# Supplementary material for: Identification of Heterotic Groups and Patterns Based on Genotypic and Phenotypic Characteristics Among Rice Accessions of Diverse Origins
Source: Front Genet. 2022 Jan 28;13:811124. doi: 10.3389/fgene.2022.811124 (PMC8832281; doi:10.3389/fgene.2022.811124)
Supplement: Supplementary file 3 [file DataSheet1.docx]

**Table S2. Over all summary on 10268 SNP dataset of 359 rice genotypes.**

| **Chromosome No.** | **SNPs No.** | **Stat Type** | **Value** |
| --- | --- | --- | --- |
| 1 | 1345 | Number of Taxa | 359 |
| 2 | 1097 | Number of Sites | 10268 |
| 3 | 1261 | Sites x Taxa | 3.6862E6 |
| 4 | 914 | Number Not Missing | 3.6862E6 |
| 5 | 814 | Number Missing | 0 |
| 6 | 899 | Number Gametes | 7.3724E6 |
| 7 | 789 | Gametes Not Missing | 7.3724E6 |
| 8 | 672 | Proportion Gametes Not Missing | 1 |
| 9 | 552 | Number of loci with multiple alleles | 788 |
| 10 | 573 | Number of biallelic loci | 9480 |
| 11 | 677 | Number Heterozygous | 55951 |
| 12 | 675 | Proportion Heterozygous | 0.01518 |
| **Total** | **10268** | Average Minor Allele Frequency | 0.21989 |

**Table S3. Distribution of various categories of breeding lines into different genetic groups.**

| **Breeding Type** | **Group 1** | **Group 2** | **Group 3** | **Group 4** | **Group 5** | **Group 6** | **Un-estimated group** | **Total** |
| --- | --- | --- | --- | --- | --- | --- | --- | --- |
| **Inbred** | 114 | 16 | 35 | - | 15 | 01 | 02 | 183 |
| **Maintainer** | 08 | 12 | 06 | 01 | - | 25 | 01 | 53 |
| **Restorer** | 39 | 03 | 31 | 40 | - | 04 | 03 | 120 |
| **TGMS line** | - | - | 01 | - | - | - | - | 01 |
| **Unknown** | - | - | 02 | - | - | - | - | 02 |
| **Total** | **161** | **31** | **75** | **41** | **15** | **30** | **06** | **359** |

**Table S4. Grouping of genotypes into different clusters, sub-groups, their frequency and percent share of each group.**

| **Cluster** | **Frequency** | **Accessions** | **Share%** |
| --- | --- | --- | --- |
| **I** | 67 | G1: C481 C234 C389 C366 C368 C290 C293 C296 C294 C288 C295 C224 C210 C289 C287 C278 C221 C226 C220 C223 C329 C332 C279 C222 C271 C333 C341 C337 C338 C357 C354 C340 C342 C334 C343 C498 C379 C380 C376 C378 C312 C286 C317 C529  G2: C269 C270 C300 C307 C310 C197 C358 C363 C304 C371 C508 C518 C520 C349 C530 C351 C531 C364 C374 C372 C369 C544 C370. | 18.66% |
| **II** | 90 | G1: C243 C258 C367 C382 C499 C503.  G2: C256 C505 C268 C493 C535 C202 C492 C512 C509 C309 C245 C308 C263 C301 C412 C491 C375 C281 C321 C319 C285 C539 C327 C297 C302 C238 C236 C325 C326.  G3: C541 C542 C207 C362 C477 C525 C524 C526 C527 C523 C339 C510 C528 C206 C356.  G4: C533 C203 C391 C502 C405 C255 C402 C252 C404 C254 C298 C537 C373 C260 C305 C536 C201 C500 C247 C399 C257 C299 C264 C323 C411 C208 C394 C240 C501 C248 C395 C497 C241 C400 C249 C396 C242 C532 C266 C209. | 25.07% |
| **III** | 05 | C235 C291 C292 C377 C511. | 1.39% |
| **IV** | 38 | C401 C409 C408 C407 C273 C440 C229 C216 C272 C330 C217 C219 C478 C417 C218 C230 C212 C211 C480 C274 C233 C215 C213 C214 C225 C416 C439 C277 C227 C479 C284 C484 C485 C482 C486 C228 C418 C348. | 10.58% |
| **V** | 116 | C261 C306 C259 C468 C328 C513 C448 C192 C450 C433 C496 C455 C473 C311 C196 C451 C459 C426 C186 C283 C188 C460 C464 C452 C463 C471 C434 C195 C457 C390 C453 C458 C193 C314 C320 C413 C461 C454 C443 C415 C187 C392 C280 C313 C200 C462 C470 C441 C444 C276 C191 C495 C475 C194 C387 C429 C335 C336 C431 C385 C410 C250 C420 C421 C422 C419 C403 C253 C381 C393 C239 C384 C324 C205 C507 C435 C267 C190 C538 C199 C265 C474 C494 C386 C185 C456 C322 C423 C262 C446 C316 C516 C406 C359 C449 C445 C447 C476 C490 C540 C436 C437 C438 C244 C397 C398 C246 C275 C282 C534 C251 C303 C365 C237 C424 C465. | 32.31% |
| **VI** | 43 | C383 C522 C355 C360 C414 C432 C315 C318 C198 C515 C204 C467 C521 C469 C489 C442 C231 C232 C189 C361 C504 C506 C347 C487 C517 C543 C519 C425 C430 C428 C388 C346 C472 C345 C350 C352 C488 C344 C466 C353 C427 C331 C514. | 11.14% |
| **Total** | **359** |  | **100%** |

**Table S6. Representation of locations and breeding types in each cluster.**

| **Clusters** | |  | **I** |  |  | **II** |  |  | **III** |  |  | **IV** |  |  | **V** |  |  | **VI** |  |
| --- | --- | --- | --- | --- | --- | --- | --- | --- | --- | --- | --- | --- | --- | --- | --- | --- | --- | --- | --- |
| **Origin** | | **Inbred line** | **Restorer** | **Maintainer** | **Inbred line** | **Restorer** | **Maintainer** | **Inbred line** | **Restorer** | **Maintainer** | **Inbred line** | **Restorer** | **Maintainer** | **Inbred line** | **Restorer** | **Maintainer** | **Inbred line** | **Restorer** | **Maintainer** |
| **Fujian** | | C389 |  | C290 |  | C268 C281 C319 |  |  |  |  |  |  |  |  |  |  | C506* |  |  |
| **Guangdong** | | C210 C333 C341 C337 C338 C354 C340 C342 C334 C343 C379 C529 C197 C358 C508 C518 C520 C351 C370 | C366C498 C380 C300 C310 C372 | C481 C294 C357 C286 | C207  C362 C339 | C243 C258 C382 C499 C256 C309 C245 C308 C301 C375 C321 C539 C327 C325 C326 C533 C203 C391 C502 C405 C255 C402 C252 C404 C254 C298 C537 C373 C260 C305 C500 C247 C399 C257 C299 C264 C323 C411 C208 C394 C240 C501 C248 C395 C497 C241 C400 C249 C396 C242 C532 C266 C209 | C367 |  |  | C292 | C401 C284 | C409 C408 C407 | C273 C440 C229 C216 C272 C330 C217 C219 C478 C417 C218 C230 C212 C211 C480 C274 C215 C213 C214 C225 C416 C439 C277 C479 C484 C485 C482 C486 C228 C418 | C306 C468 C513 C448 C192 C450 C433 C496 C455 C473 C196 C451 C459 C426 C186 C283 C188 C460 C464 C452 C463 C471 C434 C195 C457 C390 C453 C458 C193 C413 C461 C454 C443 C415 C187 C392 C280 C200 C462 C470 C441 C444 C276 C191 C495 C475 C194 C387 C429 C335 C336 C431 C385 C384 C205 C507 C435 C267 C190 C538 C199 C265 C474 C494 C386 C185 C456 C423 C316 C516 C359 C449 C445 C447 C436 C275 C282 C365 C237 C424 C465 | C261 C259 C328 C311 C314 C320 C313 C410 C250 C420 C421 C422 C419 C403 C253 C381 C393 C239 C262 C406 C476 C540 C437 C438 C244 C397 C398 C246 C534 C251 C303 |  | C355 C360 C414 C432 C315 C318 C198 C515 C204 C521 C469 C489 C189 C361 C347 C487 C517 C543 C519 C425 C430 C428 C388 C346 C472 C345 C350 C352 C488 C344 C353 C427 C331 C514 |  | C442 |
| **Guangxi** | |  |  | C234C296 C295 C224 |  | C536 C201 |  |  |  |  |  |  | C233 C227 | C446 |  |  | C383 C522 C504 |  | C231 C232 |
| **Hainan** | |  |  |  |  |  |  |  |  |  |  |  |  |  |  |  | C467 C466 |  |  |
| **Heilongjiang** | |  |  |  | C524 C526 C527 |  |  |  |  |  |  |  |  |  |  |  |  |  |  |
| **Hubei** | |  |  |  | C503 |  |  |  |  |  |  |  |  | C324 |  |  |  |  |  |
| **Hunan** | | C279 | C307 | C289 | C512 C509 C541 C542 C510 | C302 C238 C236 |  |  |  |  | C348 TGMS Line |  |  | C490 |  |  |  |  |  |
| **Jiangsu** | |  |  |  | C477 C525 |  |  |  |  |  |  |  |  |  |  |  |  |  |  |
| **Jiangxi** | | C371 | C269 C270 | C293 C288 |  |  |  |  |  |  |  |  |  |  | C322 |  |  |  |  |
| **Jilin** | |  |  |  | C523 |  |  |  |  |  |  |  |  |  |  |  |  |  |  |
| **Anhui** | |  |  |  |  |  |  |  |  | C235 |  |  |  |  |  |  |  |  |  |
| **Pakistan** | |  |  |  | C356 |  |  |  |  |  |  |  |  |  |  |  |  |  |  |
| **Philippines** | | C378 C363 C304 C364 C374 | C544 |  |  |  |  | C377 |  |  |  |  |  |  |  |  |  |  |  |
| **Sichuan** | |  |  | C368C287 |  | C493 C535 C202 C492  C263 C412 C491 C285 | C297 | C511 |  | C291 |  |  |  |  |  |  |  |  |  |
| **Iran** | | C369 |  |  |  |  |  |  |  |  |  |  |  |  |  |  |  |  |  |
| **Unknown** | |  |  |  |  |  |  |  |  |  |  |  |  |  |  |  |  |  |  |
| **USA** | |  |  |  | C206 |  |  |  |  |  |  |  |  |  |  |  |  |  |  |
| **Yunnan** | | C312 C317 C349 C530 C531 |  |  |  |  |  |  |  |  |  |  |  |  |  |  |  |  |  |
| **Taibei** | | C376 |  |  |  |  |  |  |  |  |  |  |  |  |  |  |  |  |  |
| **Thailand** | |  |  |  | C528 |  |  |  |  |  |  |  |  |  |  |  |  |  |  |
| **Chongqing** | |  |  |  | C505* |  |  |  |  |  |  |  |  |  |  |  |  |  |  |
| **Zhejiang** | | C278 C221 C226 C220 C222 C223 C329 C332 | C271 |  |  |  |  |  |  |  |  |  |  |  |  |  |  |  |  |
| **Total= 359** | **42** | **11** | **14** | **19** | **69** | **02** | **02** |  | **03** | **03** | **03** | **32** | **84** | **32** |  | **40** |  | **03** |  |

*: Unknown breeding type.

**Table S7. List of commercial breeding lines included in the genotyped accessions, their mean yield performance and hybrid combinations,**

**produced across different region of south China**.

| **S.No.** | **Female parent** | **Male parent** | **A x R** | **Hybrid combinations** | **Year** | **Variety approved No.** | **Mean yield (kg/667 m2)** | **Region** |
| --- | --- | --- | --- | --- | --- | --- | --- | --- |
| 1 | Anfeng A | Yuehesimiao | 478 x 190 | Antianyouyuehesimiao | 2020 | 国审稻20206191 | 675.98 | National |
| 2 | Guang8 A | Yuehesimiao | 228 x 190 | Guang8youyuehesimiao | 2020 | 川审稻20206007 | 564.38 | Sichuan |
| 3 | Hengfeng A | Yuehesimiao | 227 x 190 | Hengfengyouyuehesimiao | 2021 | 豫审稻20210002 | 596 | Henan |
| 4 | Hengfeng A | Yuehesimiao | 227 x 190 | Hengfengyouyuehesimiao | 2019 | 粤审稻20190070 | 480.64 | Guangdong |
| 5 | Taifeng A | Yuehesimiao | 230 x 190 | Taiyouyuehesimiao | 2020 | 川审稻20206006 | 573.05 | Sichuan |
| 6 | Longtepu A | Yuehesimiao | 290 x 190 | Teyouyuehesimiao | 2020 | 粤审稻20200041 | 449.6 | Guangdong |
| 7 | Tianfeng A | R998 | 330 x 203 | Tianyou 998 | 2004 | 粤审稻2004008 | 445.6 | Guangdong |
| 8 | Tianfeng A | R998 | 330 x 203 | Tianyou 998 | 2006 | 国审稻2006052 | 512.625 | National |
| 9 | Tianfeng A | R998 | 330 x 203 | Tianyou 998 | 2005 | 赣审稻2005041 | 490.335 | Jiangxi |
| 10 | Tianfeng A | R122 | 330 x 298 | Tianyou122 | 2005 | 粤审稻2005022 | 504.05 | Guangdong |
| 11 | Tianfeng A | R122 | 330 x 298 | Tianyou122 | 2009 | 国审稻2009029 | 485.845 | National |
| 12 | Tianfeng A | R308 | 330 x 251 | Tianyou308 | 2006 | 粤审稻2006019 | 484.8 | Guangdong |
| 13 | Tianfeng A | R368 | 330 x 257 | Tianyou368 | 2005 | 粤审稻2005025 | 482.15 | Guangdong |
| 14 | Tianfeng A | R428 | 330 x 245 | Tianyou428 | 2006 | 粤审稻2006022 | 473.25 | Guangdong |
| 15 | Wufeng A | Yuehesimiao | 272 x 190 | Wuyouyuehesimiao | 2020 | 赣审稻20200029 | 618.31 | Jiangxi |
| 16 | Wufeng A | Yuehesimiao | 272 x 190 | Wuyouyuehesimiao | 2016 | 粤审稻2016014 | 491.43 | Guangdong |

**Table S8.** Mean performance of 17 parents and their F_1_ hybrids for earliness, plant stature, yield and yield related traits.

| S.No. | F1 hybrid name | Days to 50% heading | Plant height | Panicles per plant | Grains per panicle | 1000- grain weight | Grains weight per panicle |
| --- | --- | --- | --- | --- | --- | --- | --- |
| 1 | TianfengA × Guang122 | 84.33 n-q | 106.33 k-q | 8.333 b-i | 152.46 a-g | 29.27 cde | 24.50 e-n |
| 2 | TianfengA × Huanghuazhan-1 | 85.33 m-q | 103.45 n-r | 6.777 f-m | 134.18 a-k | 28.90 def | 26.21 c-j |
| 3 | TianfengA × Huazhan | 86.00 l-p | 109.11 g-p | 6.667 g-m | 127.62 b-k | 25.98 j-p | 12.59 uvw |
| 4 | TianfengA × Minghui63 | 88.67 i-m | 114.78 c-i | 7.443 d-l | 117.07 e-l | 30.74 ab | 26.74 c-i |
| 5 | TianfengA × Wushansimiao | 84.00 n-q | 107.00 i-q | 9.000 a-f | 119.35 e-l | 27.27 g-j | 25.72 c-k |
| 6 | TianfengA × Huanghuazhan | 89.67 g-k | 102.89 o-r | 5.667 k-n | 167.80 abc | 26.91 h-m | 25.89 c-j |
| 7 | TianfengA × Minhui3301 | 91.00 f-i | 115.89 c-g | 6.553 g-n | 132.97 a-k | 31.03 ab | 27.26 c-g |
| 8 | TianfengA × Chenghui727 | 89.67 g-k | 112.00 d-m | 6.110 i-n | 128.61 b-k | 30.85 ab | 24.40 e-n |
| 9 | TianfengA × Yahui2115 | 90.67 f-j | 118.33 cd | 5.553 k-n | 141.23 a-i | 28.66 d-g | 22.47 f-p |
| 10 | TianfengA × Gui99 | 85.00 n-q | 107.33 h-q | 8.667 b-g | 111.51 g-m | 28.02 e-h | 14.82 t-w |
| 11 | TianfengA × Yuexiangzhan | 86.67 k-o | 114.89 c-h | 6.333 h-n | 143.65 a-i | 27.63 f-i | 25.10 d-l |
| 12 | TianfengA × Yuenongsimiao | 87.33 j-n | 107.00 i-q | 7.220 e-m | 150.52 a-g | 25.71 l-r | 28.27 c-f |
| 13 | TaifengA × Guang122 | 76.33 r | 101.11 qrs | 7.110 f-m | 114.12 f-l | 26.53 i-n | 35.25 ab |
| 14 | TaifengA × Huanghuazhan-1 | 86.00 l-p | 106.56 k-q | 8.447 b-h | 114.32 e-l | 25.97 j-p | 30.82 bcd |
| 15 | TaifengA × Huazhan | 82.33 q | 105.67 l-r | 8.003 c-j | 114.34 e-l | 26.78 h-m | 21.28 i-s |
| 16 | TaifengA × Minghui63 | 83.33 o-q | 110.33 e-o | 9.443 a-e | 104.28 h-n | 30.29 bc | 31.54 bc |
| 17 | TaifengA × Wushansimiao | 76.67 r | 106.67 j-q | 9.000 a-f | 116.54 e-l | 25.67 l-r | 9.48 w |
| 18 | TaifengA × Huanghuazhan | 84.67 n-q | 101.22 qrs | 8.110 c-j | 95.74 j-n | 25.54 m-s | 26.16 c-j |
| 19 | TaifengA × Minhui3301 | 87.33 j-n | 115.89 c-g | 8.443 b-h | 122.29 d-k | 29.93 bcd | 24.88 e-m |
| 20 | TaifengA × Chenghui727 | 87.33 j-n | 118.00 cde | 8.333 b-i | 98.73 i-n | 31.29 ab | 19.98 k-t |
| 21 | TaifengA × Yahui2115 | 85.67 m-q | 117.67 c-f | 6.887 f-m | 130.43 a-k | 27.95 e-h | 27.14 c-h |
| 22 | TaifengA × Gui99 | 83.67 opq | 104.89 l-r | 8.667 b-g | 114.70 e-l | 27.17 h-k | 36.48 ab |
| 23 | TaifengA × Yuexiangzhan | 85.33 m-q | 107.33 h-q | 9.667 a-d | 120.96 d-l | 26.37 i-o | 24.78 e-n |
| 24 | TaifengA × Yuenongsimiao | 83.00 pq | 104.33 m-r | 6.333 h-n | 159.16 a-e | 25.38 o-s | 24.05 e-o |
| 25 | Guang8A × Guang122 | 93.00 d-g | 108.33 g-q | 7.000 f-m | 137.11 a-j | 24.27 s-v | 19.53 l-t |
| 26 | Guang8A × Huanghuazhan-1 | 92.67 d-h | 112.22 d-l | 9.777 abc | 102.46 i-n | 25.93 j-p | 21.48 g-s |
| 27 | Guang8A × Huazhan | 89.67 g-k | 101.55 p- s | 5.113 mn | 158.76 a-f | 21.92 xza | 20.77 j-s |
| 28 | Guang8A × Minghui63 | 94.67 cde | 114.56 c-i | 7.000 f-m | 125.84 c-k | 26.49 i-o | 22.06 g-q |
| 29 | Guang8A × Wushansimiao | 90.67 f-j | 108.22 g-q | 6.667 g-m | 123.14 c-k | 24.27 s-v | 18.99 n-t |
| 30 | Guang8A × Fuhui676 | 101.67 b | 127.33 ab | 5.220 l-n | 140.30 a-j | 25.80 k-r | 23.79 e-o |
| 31 | Guang8A × Huanghuazhan | 92.00 e-i | 108.55 g-q | 7.447 d-l | 141.24 a-i | 21.95 xza | 28.33 cde |
| 32 | Guang8A × Minhui3301 | 94.67 cde | 115.11 c-h | 8.000 c-j | 102.23 i-n | 25.23 n-s | 19.89 k-t |
| 33 | Guang8A × Chenghui727 | 93.00 d-g | 110.00 f-o | 7.443 d-l | 120.55 d-l | 26.27 i-o | 25.73 c-k |
| 34 | Guang8A × Yahui2115 | 91.67 e-i | 120.89 bc | 6.000 j-n | 165.18 a-d | 25.14 o-s | 23.91 e-o |
| 35 | Guang8A × Gui99 | 91.00 f-i | 110.78 d-n | 6.890 f-m | 138.90 a-j | 24.48 q-u | 17.29 p-v |
| 36 | Guang8A × Ce64 | 94.00 def | 109.55 g-o | 7.337 e-m | 147.77 a-h | 21.69 yza | 23.07 e-p |
| 37 | Guang8A × Yuexiangzhan | 92.33 d-h | 118.56 cd | 5.890 j-n | 137.61 a-j | 25.57 l-s | 23.06 e-p |
| 38 | Guang8A × Yuenongsimiao | 93.67 c-f | 110.11 f-o | 5.890 j-n | 173.61 a | 23.01 v-y | 40.35 a |
| 39 | Ce64 | 92.67 d-h | 92.11 tu | 11.00 a | 90.62 k-n | 20.89 z | 22.90 e-p |
| 40 | Minghui63 | 101.67 b | 109.89 f-o | 6.890 f-m | 30.41 o | 25.87 k-q | 18.34 o-u |
| 41 | Yuenongsimiao | 91.67 e-i | 107.33 h-q | 5.670 k-n | 172.21 ab | 23.59 t-w | 16.46 q-v |
| 42 | Fuhui676 | 101.67 b | 131.00 a | 6.333 h-n | 68.68 mno | 25.83 k-r | 15.66 s-v |
| 43 | Chenghui727 | 96.67 c | 114.45 c-j | 6.000 j-n | 101.26 i-n | 31.25 ab | 17.30 p-v |
| 44 | Yuexiangzhan | 95.67 cd | 114.00 c-k | 5.333 lmn | 129.03 a-k | 23.65 t-w | 24.55 e-n |
| 45 | Huanghuazhan | 95.00 cde | 106.22 k-q | 6.333 h-n | 104.13 h-n | 32.01 a | 14.68 t-w |
| 46 | Minhui3301 | 105.33 a | 109.67 g-o | 4.333 n | 62.82 no | 25.37 n-s | 23.77 e-o |
| 47 | Guang122 | 90.00 g-k | 93.78 su | 10.447 ab | 76.60 l-n | 24.73 p-t | 22.71 e-p |
| 48 | Huanghuazhan | 91.67 e-i | 103.00 n-r | 8.000 c-j | 126.51 c-k | 23.55 t-w | 21.07 i-s |
| 49 | Wushansimiao | 89.33 h-l | 114.44 c-j | 9.003 a-f | 113.34 g-m | 23.24 u-x | 12.34 vw |
| 50 | Gui99 | 91.00 f-i | 110.44 e-o | 7.777 c-k | 117.00 e-l | 27.47 g-i | 16.09 r-v |
| 51 | Huazhan | 91.00 f-i | 105.44 l-r | 8.447 b-h | 111.95 g-m | 21.53 za | 26.90 c-i |
| 52 | Yahui2115 | 95.00 cde | 128.33 ab | 6.333 h-n | 104.64 h-n | 26.89 h-m | 21.80 g-r |
| 53 | TianfengB | 71.33 s | 88.00 u | 8.780 a-g | 113.91 f-l | 26.95 h-l | 21.39 h-s |
| 54 | TaifengB | 78.67 r | 98.11 rst | 9.667 a-d | 89.45 k-n | 24.47 r-u | 21.33 h-s |
| 55 | Guang8B | 91.00 f-i | 106.22 k-q | 8.557 b-h | 113.91 f-l | 22.43 w-z | 19.09 m-t |
|  | **HSD** | **3.6118** | **7.8731** | **2.2502** | **45.001** | **1.391** | **5.847** |
|  | **Means** | **89.53** | **109.58** | **7.41** | **121.34** | **26.21** | **22.92** |
|  | **Minimum** | **71.33** | **88.00** | **4.33** | **30.41** | **20.89** | **9.48** |
|  | **Maximum** | **105.33** | **131.00** | **11.00** | **173.61** | **32.01** | **40.35** |
